# The decline of farmland birds in Spain is strongly associated to the loss of fallowland

**DOI:** 10.1038/s41598-019-45854-0

**Published:** 2019-07-01

**Authors:** Juan Traba, Manuel B. Morales

**Affiliations:** 1 Terrestrial Ecology Group, TEG-UAM, Department of Ecology, Universidad Autónoma de Madrid, Darwin, 2, E-28049 Madrid, Spain; 2 Centro de Investigación en Biodiversidad y Cambio Global (CIBC-UAM), Universidad Autónoma de Madrid, Darwin, 2, E-28049 Madrid, Spain

**Keywords:** Agroecology, Conservation biology

## Abstract

Farmland bird populations have strongly declined across Europe over the last decades due to agriculture intensification, despite successive reforms of EU’s Common Agricultural Policy (CAP). In parallel, CAP has led to a reduction of fallow land, a critical habitat for biodiversity in agroecosystems. Fallow land in Spain, a country harboring the largest European populations of many endangered farmland birds, has decreased by 1.1 million ha in 15 years. The significant positive relationship between yearly change rates of the Spanish Farmland and Cereal Bird Indices (FBI and CBI) and fallow surface change highlights the adequacy of fallow land cover as an indicator of the state of farmland bird communities at country level. Moreover, the strong and positive association between the reduction in abundance of the fallow specialist little bustard and fallow surface suggests a potential causal link between these two factors. These results highlight the need for a new CAP that guarantees the maintenance of fallow land in European agroecosystems if farmland bird populations are to be conserved.

## Introduction

Farmland is the most important habitat for bird conservation in Europe, harbouring more than 50% of bird species in the European Union (EU) and 55% of European bird species listed in the IUCN Red List^1,2^. Moreover, European farmland birds are used as general indicators of the quality of agricultural habitats for biodiversity through an official agri-environmental indicator, the Farmland Bird Index (FBI)^3^.

Agriculture intensification is the main driver of the current biodiversity loss in Europe^4^ and considered to be the major cause of farmland bird declines across the continent^2^. Agriculture intensification is a multifactorial process acting at field and landscape level^4,5^, one of whose main consequences is the loss of environmental heterogeneity at different spatial scales^5,6^. At field scale, yield and revenue maximization has led to an increase of inputs and agrarian operations (e.g. soil disruption though ploughing) that have severely reduced local biodiversity^7,8^, including arable plants, invertebrates and birds^9^. This process has also led to the loss of semi natural elements of fields and their close neighbourhood (e.g. field margins), further contributing to biodiversity declines^7,8^. At landscape scale, land consolidation and disappearance of yearly (or longer) crop rotation have favoured landscape simplification and homogenization, through the loss of non-cultivated elements (e.g. margins, hedges, fallow and wasteland), further reducing habitat availability for wildlife^10–12^. EU’s Common Agricultural Policy (CAP) has been one of the main drivers of agriculture intensification in Europe^13^, promoting landscape homogenisation, increased use of agrochemicals and the abandonment of less productive fields^4,5^, despite some efforts to reverse the biodiversity loss through the application of agri-environmental schemes (AES)^14^. Moreover, AES have been only partially successful due to unclear objectives, design deficiency and low uptake^15^, and different global efficiency evaluations have yielded mixed results^16,17^. In synthesis, agriculture intensification can be considered the major cause of farmland bird and other taxa declines across the continent^2,4,6^.

**Figure 1:**
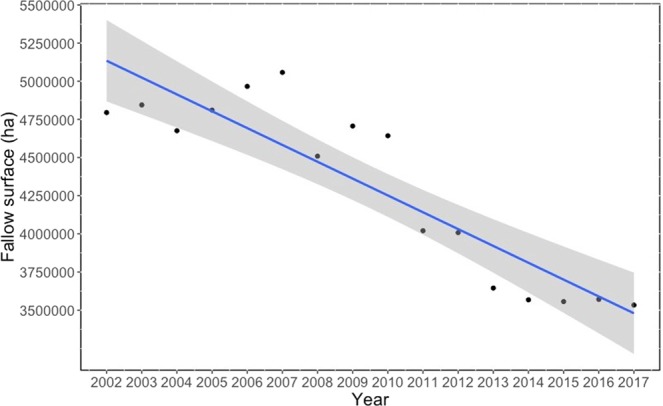
Temporal trend in the surface occupied by fallow land in Spain (2002–2017). The linear regression line is shown in blue, and 95% Confidence Intervals in grey (linear regression: adjusted R^2^ = 0.800; p < 0.0001).

The proportion of fallow land can be used as a measure of landscape scale heterogeneity and thus of agriculture intensification^18,19^. Fallow land is the cultivated land that is not seeded for one or more growing seasons^20^. Thus, fallows include different semi-natural grasslands and pastures that will eventually be ploughed for a new crop cycle^19^. Adequately managed, fallows are one of the most important habitats for wildlife, and particularly for farmland birds, in agricultural landscapes, due to the high diversity and abundance of food resources that they provide such as weeds, seeds, and invertebrates, as well as vegetation cover for foraging or nesting^6,21^. In Spain and other Mediterranean countries, fallows have been crucial for the maintenance of farmland biodiversity^18,22^. In spite of its relevance for wildlife and of more than two decades of EU’s agri-environmental measures aiming at preserving farmland biodiversity, the surface of fallow land has significantly decreased in Spain during the same period (Fig. 1), implying a loss of 1.1 million ha. Such decrease has been specially marked after 2008, when EU regulations put an end to farmers’ obligation to keep fallow 10% of their land. In neighbouring Portugal, fallows have decreased by 24.4% in only three years, between 2013 and 2016^23^.

In the Iberian Peninsula, dry cereal farmland, also known as cereal-steppe^22^, is the dominant agricultural habitat and hosts important populations of threatened steppe and farmland birds, including species whose European or world strongholds are found in Iberia^22^. Overall, more than 80% of steppe bird species show an unfavourable conservation status in Europe^1^.

Parallel to the decline of fallows, the populations of most of Spanish farmland bird species have declined during the 1990–2000 period^1^ and afterwards^24^. A species of particular conservation concern is the little bustard *Tetrax tetrax*, an endangered steppe bird included in the Annex I of EU’s Birds Directive whose populations have declined by 50% between 2005 and 2016^25^. The little bustard is a fallow specialist linked to cereal steppes^21,26,27^ whose negative trend in Spain between 1998 and 2017 - based on data from the Spanish Common Bird Monitoring Program (CBMP)^24^ - could be associated to the negative trend of fallow surface over the same period.

Declines are similar or even stronger in other globally threatened farmland birds^24^. These bird declines could also be associated with the decrease of fallow surface in Spain, but information on this regard is lacking. The aim of this work is to test the relationship between farmland bird trends and changes in fallow surface in Spain. More specifically, we examine the relationship between changes in fallow land and the change rate in the population index of a fallow specialist, the little bustard, and the general FBI for Spain. Additionally, we examine the relationship of changes in fallow land with a combined population index of those species considered as cereal specialist (i.e. more abundant in cereal farmland than in other agricultural landscapes) under the Spanish CBMP (the cereal bird index, CBI) in order to assess the potential impact of fallow loss in the most extended agricultural system of the country^28^. According to the habitat relationships of these species described in the literature, we expect a close association of changes in the two combined indices considered (FBI and CBI), as well as the little bustard population index, with fallow trends.

## Results

**Figure 2:**
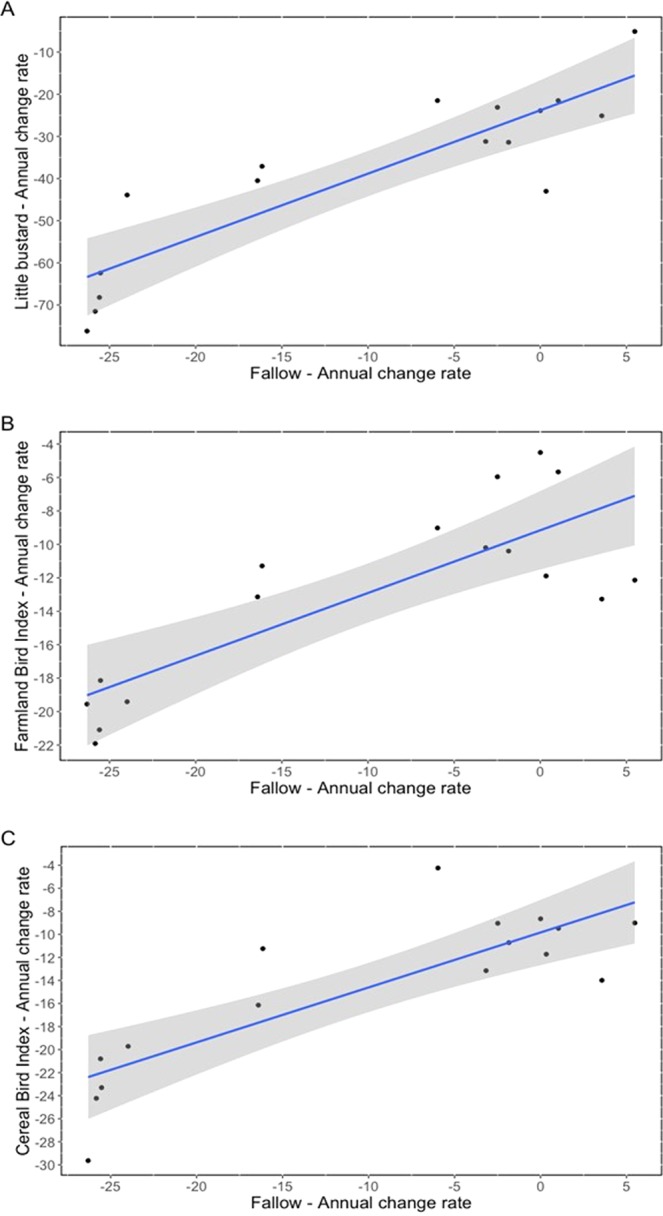
Relationship between species and fallow land annual change in Spain between 2002 and 2017. (**A**) Little bustard (adjusted R^2^ = 0.761; p < 0.00001). (**B**) Farmland birds (adjusted R^2^ = 0.644; p < 0.001). (**C**) Cereal birds (adjusted R^2^ = 0.668; p < 0.001). Linear regression lines are shown in blue, and 95% Confidence Intervals in grey.

In Spain, young and old fallows significantly decreased since 2002 (Fallows: −16.1%; Old Fallows: −41.8%; Fig. 1). The change rate of the little bustard population index during the period 2002–2017 was strongly correlated with the change rate of fallow surface over the same period (adjusted R^2^ = 0.761; p < 0.0001; Fig. 2A). When the change rate of the Spanish FBI was considered, the association with the fallow surface trend was again strongly significant (adjusted R^2^ = 0.644; p < 0.001; Fig. 2B). Finally, when the change rate in the Spanish CBI was considered, the association with fallow trend was even more significant (adjusted R^2^ = 0.668; p < 0.001; Fig. 2C).

## Discussion

These results indicate a nation-wide relationship between the temporal trend in the amount of a particular habitat type linked to extensive agriculture, that is fallow land, and the population trends of farmland birds. The relationship is highly explicative for the global Spanish farmland bird and the cereal specialist indices, and particularly for the fallow specialist and strongly declining little bustard. The decline is especially pronounced when the yearly change of fallows falls below −20% and the values of the three indices dramatically collapse.

It is important to bear in mind, however, that this assessment considered only trends in fallow surface, but not changes in fallow management. Unfortunately, available official statistics on this regard are fragmentary and do not cover the entire period considered in our analysis, although they clearly indicate a trend to increasing field-level intensification. Overall pesticide sales in Spain increased by 5% between 2011 and 2016^3^, and commercialization of herbicides and fungicides increased by 16.2% and 21.2%, respectively, between 2011 and 2017^28^. Similarly, the use of Nitrogen mineral fertilizers increased in the country from 55.0 kg/ha to 63.6 kg/ha on average between 2011 and 2016, although that of Phosphorus slightly declined from 11.2 kg/ha to 10.7 kg/ha in the same period^3^. In parallel, direct seeding or no-till farming, an agrarian technique with minimum soil disturbance but using herbicide for weed control, has significantly increased by 122.6% between 2008 and 2016, while the surface of non-managed fallows has decreased by 35.62% in the same period^28^. These management changes are expected to have important negative effects on biodiversity, including bird species^4^. Nevertheless, the decline in fallow surface is not the only large scale land-use change occurring in Spain in this period. More specifically, irrigated woody crops (i.e. olive groves and vineyards), which are largely unsuitable for farmland birds^22^ have increased by 105% between 2004 and 2017, yielding a surface gain of ca. 416,000 ha^5,28^.

In any case, our assessment of bird trends in relation to fallow surface shows how a widely recognized and large-scale indicator of agriculture intensification like fallow land cover^19^ and an official EU indicator of the general environmental status of farmland like the FBI can be inter-related to assess the overall health of a country’s agroecosystems. This is corroborated by the subset of species more clearly dependent on cereal farmland, the agricultural system where fallows have traditionally been more important due to crop rotation^18,19^. In addition, cereal farmland is being particularly affected by the mentioned growth of intensive woody crops. These conclusions were consistently reinforced when we examined the trend of the little bustard, a fallow specialist linked to cereal farmland^26,27^.

Fallows are a key component of farmland heterogeneity in the Iberian Peninsula^18,19,29^. At landscape scale, fallows provide a habitat that most farmland birds use complementarily to others (e.g. cereal crops, wastelands) for different vital functions such as foraging, mating or nesting^29,30^. Moreover, in intensive farmland, fallows can be the only habitat where limiting resources like food or adequate nesting sites are found (Moreira *et al*.^30,31^, Morales *et al*.^21,32^). At the field scale, adequately managed fallows can fulfil most requirements of fallow specialists, which benefit from their heterogeneous vegetation structure. For example, little bustard females require more vegetation cover for nesting, but a relatively open structure at certain height for anti-predator survey, while males select lower vegetation cover to perform their sexual display^21^. However, fallows are highly variable in their structure depending on their age, local soil conditions, water availability and management, among other factors^33^. As a consequence, not all fallows are equally suitable for the different species, which means that at large spatial scales such diversity of fallow characteristics should be promoted to maintain rich farmland bird communities^31^. In this context, not only the loss of fallows in cereal farmland, but also the intensification of their management, which leads to bare and resource-depleted ploughs, or heavily herbicide-treated no-till fields, poses a serious threat to farmland bird populations and helps understand our results on the combined indices used.

The trends in fallow surface and management discussed here are consistent with the multiscale process of agriculture intensification^4^, producing a loss of landscape heterogeneity and thus habitat availability for the different species^10–12,34^, along with a depletion of key resources due to field-level management^7,8^ Therefore, the large-scale association of Spanish farmland bird trends with the loss of fallow land highlights the urgent need to reverse the current trend to a highly intensive agriculture in Spain if the European and even world-level population strongholds of many steppe birds are going to be conserved. To that purpose, the upcoming generation of EU agri-environmental schemes to be implemented under CAP after 2020 should encourage the maintenance of fallow land. Among the tools that CAP might incorporate would be conditioning the reception of subsidies by farmers to leaving fallow a minimum surface of the land they manage. Moreover, a return to the obligation to keep at least 10% of their land as fallow may help restore the conditions previous to 2008. At the same time, in order to guarantee ecologically functional fallows in sustainable and environmentally healthy agricultural landscapes in Europe, field-level intensification should be discouraged with the aim of reducing agro-chemical inputs. To that aim, ambitious regulations addressing all levels of the food production chain up to the consumer are required.

## Methods

### Estimation of trends in fallow surface

Data were obtained from the National Survey on Agrarian Surfaces (ESYRCE), of the Spanish Ministry of Agriculture, Fisheries and Food MAPA^28^, for the period 2002–2017. These statistics are collected for all the country’s agrarian districts, although for public use they are scaled up to the province level. Therefore, they synthesize country-wide exhaustive information on agrarian land use change. We merged surface (ha) of young and old fallows in a single variable; that is, unsown fields lasting for 1 (young) or more years (old), but always within the rotation cycle. Then, to estimate the overall annual rate of change in fallow surface in Spain, we calculated a yearly rate of change (%) since 2002, this year considered as 0.

### Estimation of change rate in bird populations

We used bird data from the CBMP in Spain (SACRE Program), which is active since 1998. However, since fallow surface data were available only from 2002 onwards, we selected data for the period 2002–2017^35^. SACRE comprises data from censuses carried out by volunteers in a set of UTM 10 × 10 km cells distributed across the country (see Supplementary Fig. 1), which are sampled every year, following the same standard methodology^35^. From these census data, SEO/BirdLife provides a bird population abundance index for each species and year, estimated using the Trend and Indices for Monitoring data (TRIM) software by fitting log-linear regression models to count data with Poisson error terms^36^. From this index, an annual change rate is estimated for each species, in a way analogous to that described for fallow surface. Therefore, the change rate of the TRIM population index can be used as a country-wide estimate of annual changes in the abundance of a species or group of species^37^. We used data for the Farmland Bird Index (FBI) in Spain, a summary population index that includes information from the species classified as common farmland birds under the Spanish CBMP (see Table S1). The FBI is an official indicator of the quality of EU’s agroecosystems for biodiversity, as well as of the effectiveness of agri-environmental measures applied under European CAP^3^. The combined population index for the subset of common farmland birds that are particularly abundant in cereal farmland (Cereal Bird Index, CBI), provided also by SEO/BirdLife, was used to explore the relationship of cereal farmland specialists with the variation in fallow surface. Finally, we used the little bustard population index, as an indicator of the response of fallow specialists to changes in fallow surface.

### Analyses

To estimate the relationship between bird trends (Little Bustard index, FBI and CBI) and fallow land, we fitted single linear regressions between change rates of bird population indices and the change rate in total fallow surface over the period considered.

## Supplementary information

The decline of farmland birds in Spain is strongly associated to the loss of fallowland

## Data Availability

Data would be freely available upon ms acceptance.
