# Supplementary material for: The decline of farmland birds in Spain is strongly associated to the loss of fallowland
Source: Sci Rep. 2019 Jul 1;9:9473. doi: 10.1038/s41598-019-45854-0 (PMC6603185; doi:10.1038/s41598-019-45854-0)
Supplement: Supplementary file 1 — The decline of farmland birds in Spain is strongly associated to the loss of fallowland [file 41598_2019_45854_MOESM1_ESM.pdf]

The decline of farmland birds in Spain is strongly associated to the loss of fallowland

Juan Traba[1,2,+], Manuel B. Morales[1,2,*,+]

[1] Terrestrial Ecology Group, TEG-UAM. Department of Ecology. Universidad Autónoma de Madrid. Darwin, 2. E-28049, Madrid, Spain.

[2] Centro de Investigación en Biodiversidad y Cambio Global (CIBC-UAM). Universidad Autónoma de Madrid. Darwin, 2. E-28049, Madrid, Spain.

* Correspondence to: manuel.morales@uam.es

[+] Both authors contributed equally

Additional Information

Table S1. List of species included in the Farmland Bird Index for Spain. English and Latin names, and trend in Spain are provided.

Common name	Latin name	Trend in Spain
European bee-eater	*Merops apiaster*	Moderate decline
Eurasian hoopoe	*Upupa epops*	Stable
Skylark	*Alauda arvensis*	Moderate decline
Calandra lark	*Melanocorypha calandra*	Moderate decline
Common kestrel	*Falco tinnunculus*	Moderate decline
Zitting cisticola	*Cisticola juncidis*	Moderate increase
Common quail	*Coturnix coturnix*	Moderate decline
Crested lark	*Galerida cristata*	Moderate decline
Black-eared wheatear	*Oenanthe hispanica*	Moderate decline
Corn bunting	*Emberiza calandra*	Stable
Spotless starling	*Sturnus unicolor*	Moderate increase
Common starling	*Sturnus vulgaris*	Stable
Black-bellied sandgrouse	*Pterocles orientalis*	Moderate decline
Barn swallow	*Hirundo rustica*	Moderate decline
House sparrow	*Passer domesticus*	Moderate decline
Eurasian tree sparrow	*Passer montanus*	Moderate decline
Spanish sparrow	*Passer hispaniolensis*	Moderate increase
Western jackdaw	*Corvus monedula*	Moderate decline
European goldfinch	*Carduelis carduelis*	Stable
Little owl	*Athene noctua*	Moderate decline
Common linnet	*Carduelis cannabina*	Stable
Red-legged partridge	*Alectoris rufa*	Moderate decline
Little bustard	*Tetrax tetrax*	Moderate decline
Greater short-toed lark	*Calandrella brachydactyla*	Moderate increase
European turtle dove	*Streptopelia turtur*	Moderate decline
Eurasian magpie	*Pica pica*	Moderate decline

Table S2. List of species included in the Cereal Bird Index for Spain. English and Latin names, and trend in Spain are provided.

Common name	Latin name	Trend in Spain
Skylark	*Alauda arvensis*	Moderate decline
Calandra lark	*Melanocorypha calandra*	Moderate decline
Lesser kestrel	*Falco naumanni*	Moderate increase
Common kestrel	*Falco tinnunculus*	Moderate decline
White stork	*Ciconia ciconia*	Moderate increase
Zitting cisticola	*Cisticola juncidis*	Moderate increase
Common quail	*Coturnix coturnix*	Moderate decline
Crested lark	*Galerida cristata*	Moderate decline
Black-eared wheatear	*Oenanthe hispanica*	Moderate decline
Corn bunting	*Emberiza calandra*	Stable
Black-bellied sandgrouse	*Pterocles orientalis*	Moderate decline
Red-legged partridge	*Alectoris rufa*	Moderate decline
Little bustard	*Tetrax tetrax*	Moderate decline
Greater short-toed lark	*Calandrella brachydactyla*	Moderate increase

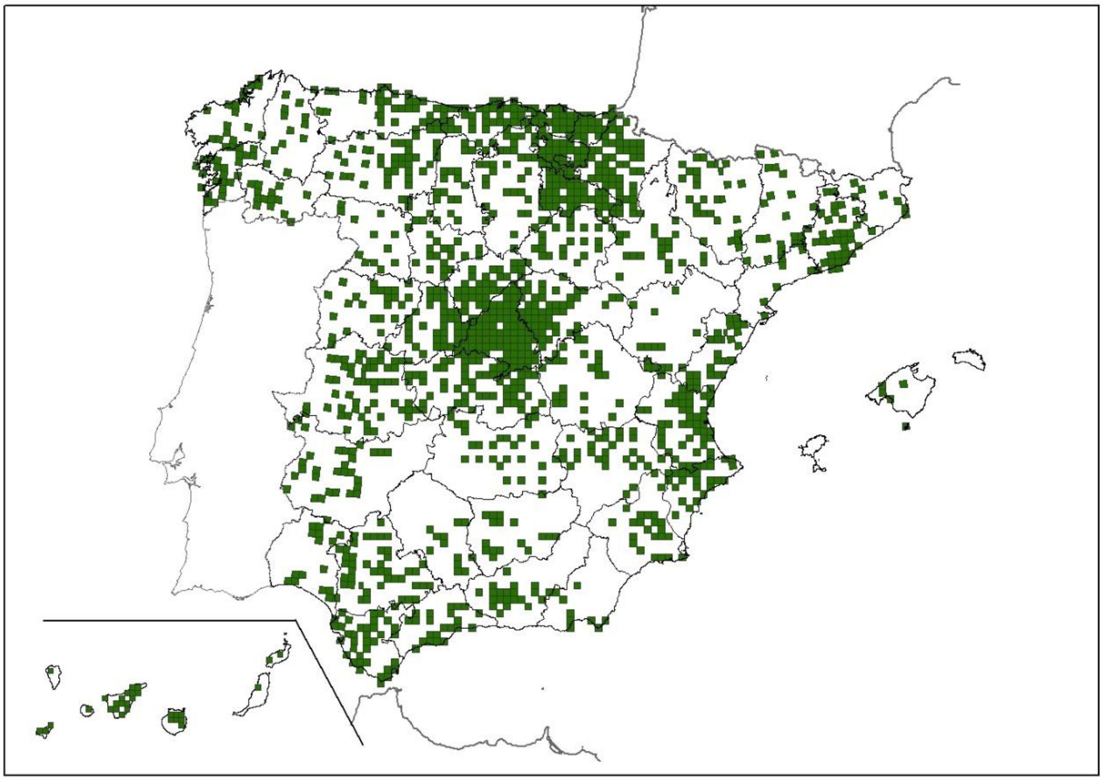

Figure S1. Map showing all 10x10 UTM squares surveyed under the Spanish Common Bird Monitoring Program (SACRE) between 1998 and 2017
